# Ipilimumab (Immune Checkpoint Inhibitors) Hypophysitis

**DOI:** 10.5334/jbsr.2161

**Published:** 2020-11-18

**Authors:** Margot Stagnetto, Patrick Mailleux

**Affiliations:** 1 UCL, BE

**Keywords:** MRI, Autoimmune, hypophysitis, immune checkpoint inhibitors

## Abstract

**Teaching Point:** Hypophysitis is a side effect of Ipilimumab that should not be confused with other conditions on imaging, such as metastasis.

## Case

**Figure 1:**
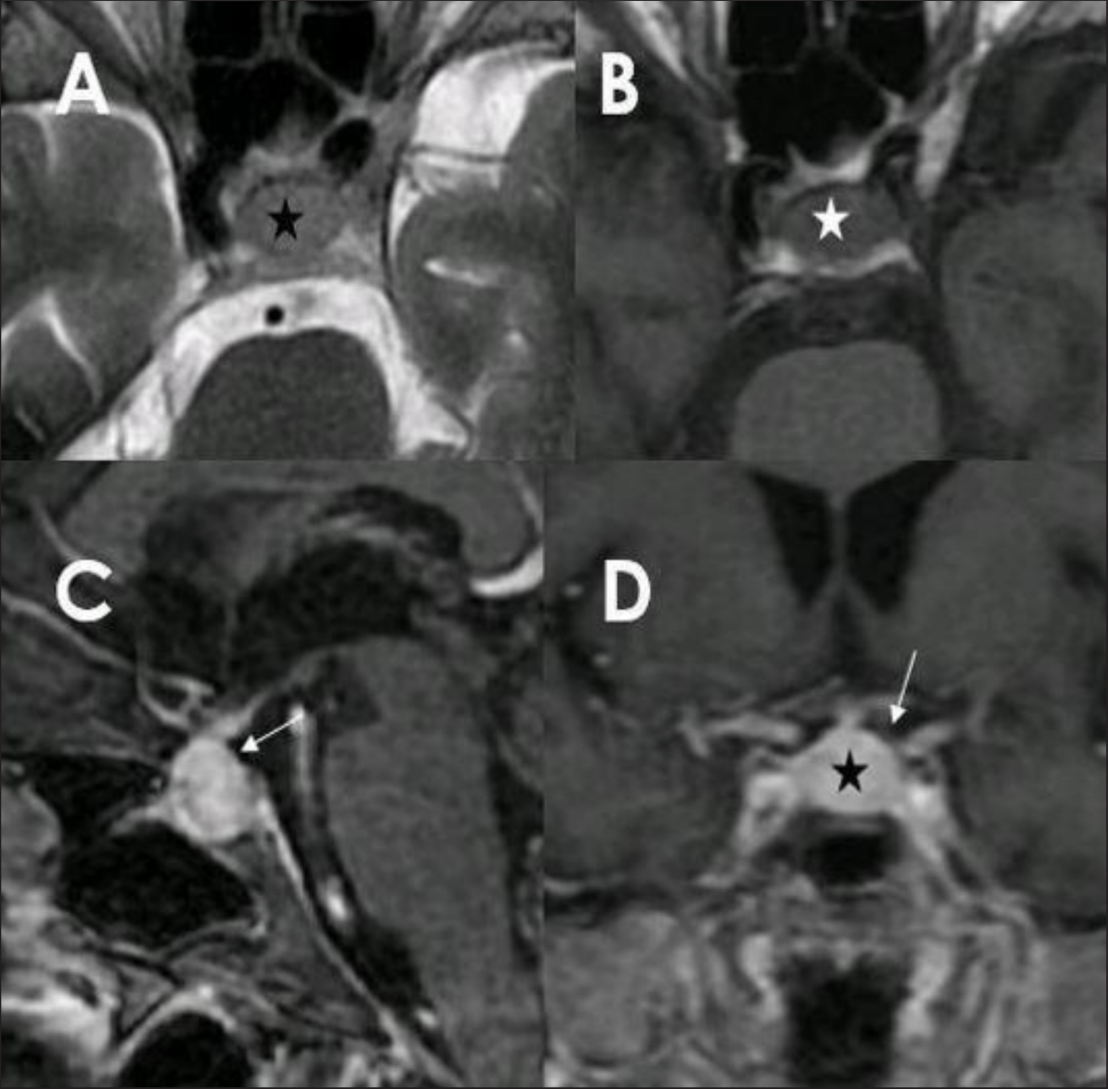


A 68-year-old female patient with history of right nephrectomy six years ago for clear cell carcinoma later developed metastases to the breast, the lung, the pancreas, treatead with nivolumab and ipilimumab, that are immune check points inhibitors. She presented with unusual severe frontal headache associated to ankle cellulitis and septic shock due to hypocorticism. She had low serum levels of potassium, T3, and cortisol. Pituitary magnetic resonance imaging (MRI) (Figure 1A–1D) showed enlarged gland (star), iso-intense on T1-weighted imaging (Figure 1B) and slightly hyperintense on T2-weighted imaging (Figure 1A), homogeneous and hyperintense after gadolinium injection. There was a non nodular stalk thickening (Figure 1C and 1D). The gland had 13 mm of height while it was 5 mm on a previous survey computed tomography. Given the treatment with Ipilimumab for four months, the differential diagnosis was metastasis versus hypophysitis. In favour of the latter are the symmetric and homogenous precontrast signal, the homogenous enhancement after injection, the loss of the normal hyperintensity of the post-hypophysis on T1-weighted imaging, the absence of floor erosion and the stalk thickening. In our patient, all these findings were present, except the post-hypophysis hyperintensity on T1-weighted imaging that was not searched for, making hypophysitis the most likely diagnosis. Ipilimumab was withdrawn and high-dose corticosteroids were introduced. A control MRI two months later showed a normal size hypophysis, confirming the diagnosis.

## Discussion

Ipilimumab (YervoyR) is a monoclonal antibody from the “checkpoint inhibitor” family. Those don’t work directly on the tumor but take the brakes off an immune response that has begun but hasn’t yet been working at its full force. It has some good results, in association to other drugs, in melanoma and in advanced kidney cancer but can produce a variety of side effects including the “autoimmune ipilimumab-induced hypophysitis,” a common side effect that affects more than 10% of the treated patients. Hypophysitis can also happen, less frequently in patients treated with nivolumab, the other patient’s medication.

Patients with hypophysitis usually present with headaches and multiple endocrine deficiencies with partial or total hypopituitarism. With the increasing use of immunotherapy in cancer patients, the radiologists should be informed of the patient’s drug regimen and possible side effects [1]. When applicable, specific imaging findings should be used to avoid delay in diagnosis and help setting appropriate treatment. In this case for example, wrong diagnosis of pituitary metastasis could have lead to unnecessary brain radiotherapy.
